# Combination of sofosbuvir, pegylated-interferon and ribavirin for treatment of hepatitis C virus genotype 1 infection: a systematic review and meta-analysis

**DOI:** 10.1186/s40199-017-0177-x

**Published:** 2017-04-20

**Authors:** Fardin Dolatimehr, Hamidreza Karimi-Sari, Mohammad Saeid Rezaee-Zavareh, Seyed Moayed Alavian, Bita Behnava, Mohammad Gholami-Fesharaki, Heidar Sharafi

**Affiliations:** 1 Student Research Committee, Baqiyatallah University of Medical Sciences, Tehran, Iran; 2 Meta-analysis Study Group, Iran Hepatitis Network, Tehran, Iran; 3 Baqiyatallah Research Center for Gastroenterology and Liver Diseases (BRCGL), Baqiyatallah University of Medical Sciences, Tehran, Iran; 4 Middle East Liver Disease (MELD) Center, Tehran, Iran; 5 Department of Biostatistics, Faculty of Medical Sciences, Tarbiat Modares University, Tehran, Iran

**Keywords:** Hepatitis C, Sofosbuvir, Ribavirin, Pegylated-interferon, Meta-analysis

## Abstract

**Background:**

Hepatitis C virus (HCV) infection is an important cause of chronic liver disease which has been affected 3% of world’s population. Some studies have shown that adding Sofosbuvir (SOF), an HCV polymerase inhibitor to the conventional therapy of Pegylated-interferon (PegIFN) plus Ribavirin (RBV) can increase the rate of sustained virologic response (SVR) among HCV-infected patients. This study was conducted to determine the effect of combination therapy with PegIFN and RBV plus SOF for chronic hepatitis C genotype 1 infection using systematic review with meta-analysis.

**Methods:**

In this study, electronic databases including PubMed, Scopus, Science Direct, and Web of Science were comprehensively searched using appropriate strategies containing all related keywords of “hepatitis C”, “PegIFN”, “RBV” and “SOF”. Studies assessed the efficacy of combination therapy with PegIFN and RBV plus SOF for chronic hepatitis C genotype 1 infection were included in the meta-analysis.

**Results:**

After screening of 757 records, we included five articles with total sample size of 411 to the meta-analysis. Based on the fixed-effect model (*χ* ^2^ = 5.29, *P* = 0.26 and I^2^ = 24.4%), pooled SVR rate for treatment regimen of PegIFN and RBV plus SOF was calculated as 88.54% (95% CI = 85.77%–91.32%).

**Conclusions:**

Combination therapy with PegIFN and RBV plus SOF results in high treatment response in patients with HCV genotype 1 infection.

**Electronic supplementary material:**

The online version of this article (doi:10.1186/s40199-017-0177-x) contains supplementary material, which is available to authorized users.

## Background

Hepatitis C infection is the major cause of acute and chronic hepatitis globally. Based on the World Health Organization (WHO) estimation, about 3% of world population is infected with hepatitis C virus (HCV) and the worldwide prevalence of chronic hepatitis C is more than 180 million who are at the predisposing to cirrhosis and/or liver cancer [1–3].

Treatment of chronic hepatitis C comprises several components including, reduction of inflammation, prevention of fibrosis, cirrhosis and hepatocellular carcinoma and virus eradication. Currently, sustained virologic response (SVR) functions as the best indicator of effective treatment [4, 5]. Although treatment decisions are influenced by the HCV genotype, combination therapy in comparison with monotherapy achieved higher response rate. Adding Ribavirin (RBV) to Pegylated-interferon (PegIFN) increased SVR rate from 15 to 20% to 40%–50% in HCV genotype 1 infection. However, to reach to more than 50% SVR, combination therapy with PegIFN and RBV lost its efficacy particularly in HCV genotype 1 infection [6–8].

Sofosbuvir (SOF) is a HCV NS5B polymerase inhibitor that results in suppression of HCV replication and life cycle. Sofosbuvir as a new direct-acting antiviral agent (DAA) was approved for treatment of chronic HCV genotypes 1 to 4 infections. HCV genotype 1-infected patients should receive PegIFN, RBV and SOF for 12 weeks. From the data of trials, the latter combination therapy results in SVR12 rates of 50%–90% [9, 10].

This study set out with the aim of assessing efficacy of 12-week combination of PegIFN, RBV and SOF for treatment of patients with chronic hepatitis C infection caused by HCV genotype 1.

## Methods

### Data resources and search strategy

In this systematic review and meta-analysis, electronic databases including PubMed, Scopus, Science Direct, and Web of Science were comprehensively searched using exact and sensitive search strategies (Additional file 1: Appendix), which concentrated on each element of HCV treatment regimen PegIFN, RBV plus SOF. On the other hand, the Google scholar was searched with appropriate keywords and after finding the last related title, we continued our search for 200 serial unrelated titles. This helped us to check the sensitivity of our search strategies. Furthermore, references of the finally included papers were investigated for retrieving any missing papers. Our last search using search strategies was performed at September 02, 2015. However, we did an updated search on April, 2016 just before analysis of data.

### Inclusion and exclusion criteria

All studies reporting the rate of SVR12 after ending treatment with PegIFN, RBV plus SOF for 12 weeks in patients with HCV genotype 1 infection were included. We considered the approach of intention-to-treat in the step of data extraction. Furthermore, we excluded studies evaluating patients with history of liver transplantation, chronic hemodialysis, kidney transplantation, history of previous treatment with DAAs, HIV/HCV coinfection, and decompensated cirrhosis (Child-Pugh B and C).

### Study selection and data extraction

The PRISMA guideline for reporting of systematic review was used [11]. Two reviewers (FD and HKS) screened all the identified papers in three levels including title, abstract, and full-text, independently. Any disagreement between these two reviewers were discussed mutually and any remained discrepancies were resolved by the discussion with a third reviewer (SMA or HSH).

Following parameters were extracted from the included studies; author’s first name, history of previous treatment, publication year, country, sample size, mean/median age, gender, body mass index (BMI), HCV RNA level before treatment, rate of cirrhotic patients, polymorphism near *IFNL3* (rs12979860), and HCV subtype 1a/1b.

### Quality assessment

For evaluating risk of biases in each included clinical trial, Cochrane’s assessment tool was used [12]. These biases are random sequence generation (selection bias), allocation concealment (selection bias), blinding of participant and personnel (performance bias), blinding of outcome assessment (detection bias), incomplete outcome data (attrition), selective reporting (reporting bias), co-interventions, intention-to-treat analysis, group similarity at baseline, compliance, timing of outcome assessments and other biases. These 12 items were scored 0 if were high risk and unclear, and scored 1 if were low risk. Then, overall score ≥6 was considered as low risk for each study.

For assessing quality of the included non-randomized studies, Newcastle-Ottawa Scale (NOS) was used [13]. This tool helps to assess methodological problems regarding selection of participants, comparability of case and control groups and also ascertain of exposure and outcomes. Any disagreement in quality assessment by two above tools were resolved by mutual discussion.

### Data analysis

We used chi-square and I-squared (lies from 0 to 100%) for evaluating heterogeneity across studies’ results. *P* value less than 0.1 was considered statistically significant for chi-squared. We evaluated publication bias by Begg’s and Egger’s tests. Based on the presence or absence of heterogeneity, random- or fixed-effect model was employed for calculation of pooled SVR12 rate and 95% confidence interval (CI). All data analyses were performed using STATA 10.

## Results

### Study screening and characteristics of the included papers

**Fig. 1:**
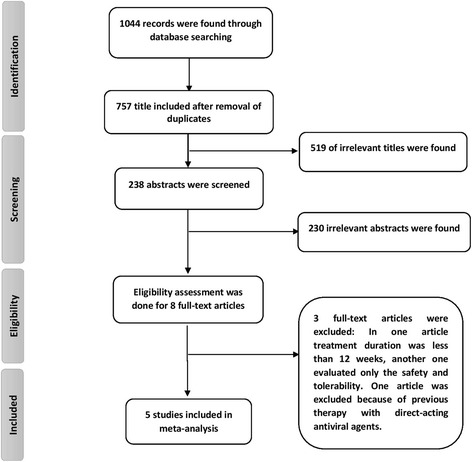
Screening of articles based on PRISMA statement

A total of 757 papers were found through database searching after removing duplications. In title screening, 519 irrelevant titles and in the abstract screening, 230 irrelevant abstracts were excluded. Then, eight full-text articles were assessed for eligibility and finally five articles with total sample size of 411 were included in our quantitative synthesis (Fig. 1).

Table 1 shows important characteristics of the included papers. They were clinical trial (*N* = 4) and cohort (*N* = 1) studies. We found three eligible studies related to 2013 and two other related to 2015. Four of our finally included studies were from the United States and another one was from Germany. Furthermore, in three of them, included patients were treatment naïve and in two other studies, included both treatment naïve and treatment experienced participants.

**Table 1:** Characteristics of the included studies^a^

First author (reference)	History of previous treatment	Publication year	Country	Study type	Sample size	Mean age (SD or range)	Male gender, n (%)	Mean BMI (SD or range)	Mean HCV RNA, log IU/mL (SD)	Cirrhosis, n (%)	rs12979860 CC/CT + TT	HCV genotype 1a/1b
Kowdley, KV [19]	TN	2013	USA and Puerto Rico	Clinical trial	52	51 (9.8)	35 (67)	27.2 (4.6)	6.5 (0.7)	7 (13.5)	0.33	0.3
Lawitz, E [20]	TN	2013	USA	Clinical trial	47	51.4 (9.4)	21 (45)	26.8 (4.5)	6.5 (0.6)	2 (4)	0.62	0.34
Lawitz, E [10]	TN	2013	USA	Clinical trial	237	51 (19–70)	209 (88)	29 (18–56)	6.4 (0.7)	54 (23)	0.41	0.29
Pearlman, BL [31]	Mix	2015	USA	Clinical trial	24	55 (ND)	15 (63)	28 (ND)	6.41 (0.59)	24 (100)	0.2	0
Steinebrunner, N [18]	Mix	2015	Germany	Cohort	51	ND	ND	ND	ND	ND	ND	ND

^a^ *Abbreviation*: *SD* Standard deviation, *TN* Treatment Naïve, *ND* Non-determined

### Quality assessment

Using Cochrane’s risk of assessment tool four clinical trials were evaluated and all of them scored more than 6 (low risk). Furthermore, using NOS, one cohort study was evaluated and this study achieved 6 out of 8 possible stars. Therefore, no study categorized as low quality and also none of them were excluded based on this assessment (Table 2).

**Table 2:** Risk of Bias assessment for the included studies

First author (reference)	Random sequence generation (selection bias)	Allocation concealment (selection bias)	Blinding of Participant and Personnel (Performance Bias)	Blinding of outcome assessment (detection bias)	Incomplete outcome data (attrition)	Selective reporting (reporting bias)	Co-interventions	Intention to treat analysis	Group similarity at baseline	Compliance	Timing of outcome assessments	Other biases	Score	Conclusion
Kowdley, KV [19]	-	-	+	+	-	-	-	-	-	-	-	-	10	Low
Lawitz, E [20]	-	-	-	-	-	-	-	-	-	-	-	-	12	Low
Lawitz, E [10]	+	+	+	+	-	-	-	-	-	-	-	-	8	Low
Pearlman, BL [31]	?	?	+	+	-	-	-	-	-	-	-	-	8	Low

### Outcome evaluation

**Fig. 2:**
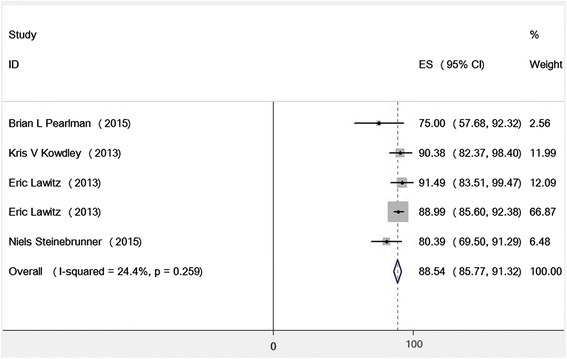
Pooled Rate of SVR for 12 weeks Treatment of SOF, PegIFN and RBV in Patients with HCV Genotype 1 Infection

There were no significant heterogeneity between results of studies based on the Chi-squared (Chi^2^ = 5.29, df = 4, *P* = 0.26) and I-squared (I^2^ = 24.4%, *P* = 0.26). Therefore, we used fixed-effect model and the pooled rate of SVR for HCV treatment regimen PegIFN, RBV plus SOF for 12 weeks was 88.54% (95% CI = 85.77%–91.32%) (Fig. 2).

We found no publication bias according to the Begg’s (*P* = 0.14), and Egger’s (*P* = 0.28) tests.

## Discussion

This study showed that combination therapy with PegIFN, RBV plus SOF with 88.5% treatment success is an effective antiviral therapy for treatment of patients with HCV genotype 1 infection. Before 2011, the standard of care for therapy of HCV genotype 1 infection was combination regimen of PegIFN and RBV for 24–72 weeks with 40–60% success and many complications [14–16]. Introduction of first DAAs in 2011, was a major step in management of patients with HCV infection and eradication of hepatitis C as a major cause of liver disease in human kind [17]. In 2013, SOF was introduced as a HCV NS5B inhibitor and approved for treatment of HCV genotype 1 infection as a combination therapy with PegIFN and RBV [10].

The PegIFN, RBV plus SOF regimen was superior to PegIFN and RBV combination therapy in terms of higher efficacy, shorter treatment duration, and fewer side-effects. Furthermore, fewer host and virus parameters affect the treatment success in comparison with PegIFN and RBV regimen [10]. Among host factors, rs12979860 and cirrhosis were the factors modified treatment response in PegIFN, RBV plus SOF regimen [10, 18, 19]. Hopefully, SOF is a DAA with high-resistance barrier and none of the included studies in this meta-analysis found the resistance-associated substitutions such as NS5B Ser282Thr in the baseline or in patients with treatment failure [10, 19, 20].

It has been proved that combination of PegIFN, RBV and SOF can lead to complete elimination of HCV RNA in about 95% of HCV-infected patients at week 4 of treatment [10, 19, 20]. Furthermore, it has been reported that both 12- and 24-week treatment regimens with PegIFN, RBV and SOF can make about 89% SVR rate and therefore they have not considerable difference in term of response to therapy [19]. As a result, it can be suggested that response-guided therapy plays no role in the treatment with PegIFN, RBV plus SOF combination therapy.

Ledipasvir/Sofosbuvir is the first IFN-free treatment regimen for HCV which was approved by FDA in 2014. It provides >95% SVR rate among non-cirrhotic HCV genotype 1 patients [21–23]. Also, because of no existence of IFN in this regimen, it has fewer adverse-events. However, this regimen is not affordable in the most of the developing countries where the frequency of HCV infection is high. Moreover, generic SOF has been produced in many countries in domestic pharmaceutical companies and available with a reasonable price which can be afforded by most of the patients. Furthermore, the most common HCV genotype worldwide is genotype 1 and therefore the evaluated regimen in our project can be used in the low- and middle-income countries regarding its high efficacy [24]. Meanwhile, other regimens containing DAAs with high efficacy have been presented including Daclatasvir/Sofosbuvir [25], Paritaprevir-r/Ombitasvir/Dasabuvir [26], Simeprevir-containig regimens [27], Grazoprevir/Elbasvir [28], and Velpatasvir/Sofosbuvir [29].

The future treatments of HCV will be IFN-free regimens with high-resistance barrier which can be applied for treatment of special group patients and can clear the virus in nearly 100% of the HCV-infected patients. With knowing that the ultimate HCV treatment will be available, there is a great concern to clear HCV infection until 2030 globally. However, there are major steps toward eradication of HCV which should be addressed in the program of HCV eradication including development of HCV vaccine and other preventive strategies, availability and affordability of high efficacious regimens in developing countries, nation-wide screening programs for finding the patients with HCV infection, and paying special attention to special patient groups such as thalassemia, kidney- and liver-transplant patients and HIV/HCV co-infected patients [17, 30].

## Conclusions

In conclusion, PegIFN, RBV plus SOF regimen is a highly effective therapy for treatment of HCV genotype 1 infection. While there are more expensive HCV antiviral treatments with higher efficacy than that of obtained by PegIFN, RBV plus SOF, the low cost of SOF and availability of this medication in many developing countries make the PegIFN, RBV plus SOF regimen as the recommended regimen for treatment of HCV genotype 1 infection especially when the patients cannot afford IFN-free regimens.

## Additional file

Additional file 1:
**Appendix.** Search strategies. (DOC 26 kb)

## Abbreviations

CI: Confidence interval
DAA: Direct-acting antiviral agent
HCV: Hepatitis C virus
NOS: Newcastle-Ottawa Scale
PegIFN: Pegylated-interferon
RBV: Ribavirin
SOF: Sofosbuvir
SVR: Sustained virologic response
