# Supplementary material for: Combination of sofosbuvir, pegylated-interferon and ribavirin for treatment of hepatitis C virus genotype 1 infection: a systematic review and meta-analysis
Source: Daru. 2017 Apr 20;25:11. doi: 10.1186/s40199-017-0177-x (PMC5397824; doi:10.1186/s40199-017-0177-x)
Supplement: Additional file 1: — Appendix. Search strategies. (DOC 26 kb) [file 40199_2017_177_MOESM1_ESM.doc]

**Appendix**

**Search Strategies**

1. ***PubMed***

*("Sofosbuvir"[Supplementary Concept] OR “Sofosbuvir”[tiab] OR Sofosbuvir*[tiab] OR Sovaldi*[tiab] OR GS-7977*[tiab]) AND ("Peginterferon alfa-2b"[Supplementary Concept] OR "Peginterferon alfa-2a"[Supplementary Concept] OR "PEG-IFN-SA"[Supplementary Concept] OR “Pegylated interferon”[tiab] OR "Peginterferon”[tiab] OR Interferon*[tiab] OR Peginterferon*[tiab]) AND ("Ribavirin"[Mesh] OR "Ribavirin"[tiab] OR Ribavirin*[tiab])*

1. ***Scopus***
2. *TITLE-ABS-KEY ("Sofosbuvir" OR Sofosbuvir* OR Sovaldi* OR GS-7977*)*
3. *TITLE-ABS-KEY("Peginterferon alfa-2b" OR "Peginterferon alfa-2a" OR "PEG-IFN-SA" OR "Pegylated interferon" OR “Peginterferon” OR “Interferon” OR Interferon* OR Peginterferon*)*
4. *TITLE-ABS-KEY ("Ribavirin" OR Ribavirin*)*
5. *#1 AND #2 AND #3*
6. *#4 AND NOT INDEX(medline)*

*(TITLE-ABS-KEY ("Sofosbuvir" OR Sofosbuvir* OR Sovaldi* OR GS-7977*)) AND (TITLE-ABS-KEY("Peginterferon alfa-2b" OR "Peginterferon alfa-2a" OR "PEG-IFN-SA" OR "Pegylated interferon" OR "Peginterferon" OR "Interferon" OR Interferon* OR Peginterferon*)) AND (TITLE-ABS-KEY ("Ribavirin" OR Ribavirin*)) AND NOT INDEX(medline)*

1. ***Science Direct***
2. *TITLE-ABS-KEY ("Sofosbuvir" OR Sofosbuvir* OR Sovaldi* OR GS-7977*)*
3. *TITLE-ABS-KEY("Peginterferon alfa-2b" OR "Peginterferon alfa-2a" OR "PEG-IFN-SA" OR "Pegylated interferon" OR “Peginterferon” OR “Interferon” OR Interferon* OR Peginterferon*)*
4. *TITLE-ABS-KEY ("Ribavirin" OR Ribavirin*)*
5. *#1 AND #2 AND #3*

*(TITLE-ABS-KEY ("Sofosbuvir" OR Sofosbuvir** *OR Sovaldi* OR GS-7977*)) AND (TITLE-ABS-KEY("Peginterferon alfa-2b" OR "Peginterferon alfa-2a" OR "PEG-IFN-SA" OR "Pegylated interferon" OR "Peginterferon" OR "Interferon" OR Interferon* OR Peginterferon*)) AND (TITLE-ABS-KEY ("Ribavirin" OR Ribavirin*))*

1. ***Web of Science***
2. *TS=("Sofosbuvir" OR Sofosbuvir** *OR Sovaldi* OR GS-7977*)*
3. *TS=("Peginterferon alfa-2b" OR "Peginterferon alfa-2a" OR "PEG-IFN-SA" OR "Pegylated interferon" OR “Peginterferon” OR “Interferon” OR Interferon* OR Peginterferon*)*
4. *TS=("Ribavirin" OR Ribavirin*)*
5. *1# AND 2# AND 3#*
